# Dorsal arthrodesis in prepubertal New Zealand white rabbits followed to skeletal maturity: Effect on thoracic dimensions, spine growth and neural elements

**DOI:** 10.4103/0019-5413.57280

**Published:** 2010

**Authors:** Federico Canavese, Alain Dimeglio, Charles D'Amato, Donatella Volpatti, Marie Granier, Marco Stebel, Fabio Cavalli, Bartolomeo Canavese

**Affiliations:** 1 Service de Chirurgie Orthopédique Pédiatrique – CHU Hôpital Lapeyronie, 371, Avenue du Doyen G. Giraud 34295 Montpellier, France; 2 Department of Pediatric Orthopedic Surgery – Shriners Hospital – 3101 SW Sam Jackson Park Road, Portland, OR 97225 USA; 3 Dipartimento di Scienze Animali – Università di Udine, Via delle Scienze, 206 – 30100 Udine, Italy; 4 Departement d'Anesthesie et Reanimation A – CHU Montpellier – 371, Avenue du Doyen G. Giraud 34295 Montpellier, France; 5 CSPA, Settore Stabulario Sperimentazione Animale – Università di Trieste, Via Valerio, 28 – 34127 Trieste, Italy; 6 Dipartimento di Diagnostica per Immagini, Struttura Complessa di Radiodiagnostica, Ospedale Maggiore, Piazza Ospedale, 1 – 34000 Trieste, Italy

**Keywords:** Dorsal arthrodesis, thorax and spine growth, dorsal root ganglia, prepubertal rabbits, skeletal maturity

## Abstract

**Background:**

Several studies have shown that severe spinal deformity and early arthrodesis can adversely affect the development of the spine and thorax by changing their shape and reducing their normal function. This article analyzes the consequences of posterior fusion on the growth of spine, thorax and neural elements in New Zealand white rabbits and compares with similar human data.

**Materials and Methods:**

The first section of the article analyzes the consequences of T1-T6 dorsal arthrodesis on the growth of the spine, sternum, thorax volume and neural elements in 12 prepubertal female New Zealand white rabbits, through a study of CT scans and histology specimens. The second part, evaluates thoracic dimensions in 21 children with spinal arthrodesis for treatment of deformity performed prior to nine years of age.

**Results:**

Dorsal arthrodesis in prepubertal rabbits changes thoracic growth patterns. In operated rabbits thoracic depth grows more slowly than thoracic width. The sternum as well as length of thoracic vertebral bodies in the spinal segment T1-T6 show reduced growth. Children undergoing spinal arthrodesis before nine years of age were noted to have shortened height, short trunk and disproportionate body habitus at skeletal maturity. Observed spine height and chest dimension values were reduced compared to the expected norms. The ratio between chest width and chest depth was below normal values.

**Conclusions:**

The first part of the study shows that thoracic dorsal arthrodesis in prepubertal New Zealand white rabbit influences thoracic, spine growth and affects the shape of pseudo unipolar neurons of the dorsal root ganglia. The second part demonstrates that children treated before nine years of age have significantly reduced spine height and thoracic dimensions. The thorax becomes elliptical as chest depth grows less than chest width. Both experimental and clinical findings contribute to explain reduced chest growth and subsequent thoracic growth disturbance in patients treated with early arthrodesis.

## INTRODUCTION

Development of the spine, thoracic cage and lungs is a complex process which requires synergy among the different anatomical elements. Deformation of the spine can adversely affect the development of the thorax by changing its shape and reducing its normal motion. These deformations, which can be lethal in the most severe cases, result from mutual interaction and influence of various skeletal and organic components of the thoracic cage and cavity. Untreated progressive early onset spinal deformity, congenital or idiopathic, has been associated with short trunk, short stature and often respiratory insufficiency. Pehrsson *et al*, found that mortality was significantly higher in infantile and juvenile scoliosis than adolescent scoliosis. They also showed that the mortality rate was higher in curves with severe magnitude than those with mild to moderate magnitude.^1^

Several experimental studies have focused on the anatomical influence of experimental arthrodesis on the growth of the spine, chest development and thoracopulmonary function. These studies have demonstrated that early arthrodesis as well as severe spinal deformities can adversely affect development of the spine and thorax, by changing their shape and reducing their normal mobility.^2^–^16^

Early-onset spinal deformities are abnormalities that appear in patients younger than five years of age and can be associated with costovertebral malformations, neuromuscular diseases, or 50-75% fusion of the thoracic spine under the age of 10.

For the past several decades, the standard treatment of early onset spinal deformities unresponsive to non-surgical treatment has been spinal fusion with the goal of arresting progression. Unfortunately, arthrodesis carried out in the thoracic spine at an early age does not address the impact of the deformity on lung parenchyma development or preservation of pulmonary function and does not always prevent deformity progression.^17^,^18^

Definitive spinal fusion can realign the trunk but when performed in very young patients, can severely affect lung development and can finally result in respiratory insufficiency. The inability of the thorax to ensure normal breathing, and the serious respiratory insufficiency that goes with it, characterise the thoracic insufficiency syndrome (TIS). The respiratory insufficiency is due to severe alterations in the gas exchange between atmospheric air and blood, which involve reduction of O_2_ and retention of CO_2_ in blood. The clinical picture, in the most serious cases, can be lethal. TIS affects patients who show complex spinal-vertebral deformities or severe dysplasia of the chest wall, as well as patients who have undergone surgery for vertebral arthrodesis under 10 years of age.^1^,^6^,^11^

Postmortem studies show that patients with early onset deformities have fewer alveoli than expected, and that emphysematous changes in existing alveoli are present. These studies suggest that mechanical compression is not responsible for reducing the number of alveoli, and that this is probably due to a premature cessation of alveolar proliferation. From zero to four years, the number of alveoli increases by a factor of 10, and the development of the bronchial tree ends around eight to nine years of age. An early onset scoliosis, therefore, adversely affects thoracic growth in the critical period of maximum ‘respiratory growth,’ which induces irreversible changes in the thoracopulmonary structure.^19^–^21^ Golberg *et al,* demonstrated that early surgery, in patients who developed scoliosis before the age of four does not modify the deformation caused by scoliosis and does not preserve the respiratory function, even when the anterior growth of the spine is arrested. They also found that spinal deformities necessitating surgery before five years of age had diminished respiratory function whereas deformities stabilized by nonoperative means or treated with surgery after 10 years of age had normal or acceptable pulmonary function.^6^ Arthrodesis is not the ideal treatment for very young patients, but is still a treatment of choice when major deformities of the spine must be addressed.

This article analyzes the consequences of selective dorsal arthrodesis of thoracic spine (T1-T6) on the growth of spine, thoracic volume and neural elements in operated and sham-operated New Zealand White rabbits, between prepubertal age and the end of somatic growth. Supplemental human data on the effect of spinal arthrodesis on thoracic and spinal dimensions in children younger than nine years of age is reported and compared to experimental findings.

## MATERIALS AND METHODS

This work has been presented in two sections. The first section analyzes the consequences of T1-T6 dorsal arthrodesis on growth of the spine, sternum, thorax volume and neural elements in 12 prepubertal female New Zealand white rabbits, through a study of CT scans and histology specimens. The second part includes supplemental human data which assessed effect of spinal fusion on thoracic dimensions in children younger than nine years of age at the time of surgery.

The experimental protocol has been set up in the Department of Pediatric Orthopedics at Lapeyronie' Hospital, Montpellier, France. It was then approved by the internal Animal Ethical Committee of the animal breeding department at CSPA of the University of Trieste, Italy, where the experimental study was conducted. Operative procedure and animal care were performed in compliance with national and international regulations (Italian regulation D. L.vo 116/1992, and EU regulation 86/609/EC). The clinical study was performed in the Department of Pediatric Orthopedics at Shriners Hospital for Children, Portland, OR (USA) and has been approved by the Institutional Review Board.

### 1. Experimental arthrodesis

Twelve female rabbits underwent surgery at the age of nine weeks. Anesthesia was administered by an intramuscular injection of two ml of two per cent xylazine and of 0.3 ml of tiletamine HCl and zolazepam HCl; supplementary skin anesthesia – once the rabbit was asleep – was obtained by a subcutaneous injection of one ml of two per cent lidocaine hydrochloride. Nine rabbits were operated with a modified “Wisconsin” technique^22^ or extra-canal dorsal vertebral arthrodesis, while three were “sham operated”. A dorsal approach of the thoracic spine was performed. Once muscular plane was reached, M. trapezius pars thoracica, M. latissimus dorsi, M. spinalis thoracis and M. longissimus lumborum et thoracis were retracted on both sides to allow a wide exposition of vertebral laminae, spinous processes, transverse processes, and facies articulares of the first eight thoracic vertebrae. Two ‘C’-shaped titanium bars, approximately eight centimeters in length and two millimeters in diameter, were positioned laterally at the base of the spinous process of the first six thoracic vertebrae. The bars were then fixed at the base of the spinous processes with three distinct metal-wire ligatures and multiple 2/0 non-absorbable ligatures. The metal wire was passed under the interspinal ligament and then tightened around the base of two contiguous spinous processes, while the non-absorbable ligature was used to further stabilize the assembly and prevent migration of the bars. The assembling carried out proved to be stable and functional for the purpose of the experiment. No attempt was made to fuse the costovertebral joints. The muscular plane and the subcutaneous plane were finally stitched with 2/0 suture, and the cutaneous plane with 3/0 suture, both absorbable (Vicryl, Ethicon Inc.). A sterile dressing was positioned on the surgical wound. The operation lasted an average of approximately 40 minutes for each rabbit. The identification of the subjects was always possible due to the microchips implanted under the skin. To relieve pain, 5 mg/kg of carprofen were administered subcutaneously twice daily over five days. Prevention, in the week following surgery, was pursued with an intramuscular injection of 5 mg/kg of enrofloxacin twice daily. During the experiment, three thoracic-vertebral CT scans were performed 10 (t1), 55 (t2), and 139 (t3) days after surgery with a 16-slice CT scanner (Aquilon 16, Toshiba Medical Corporation). Tomography examinations were carried out under general anesthesia, and the animals were kept supine and in specially prepared plastic basins.

The study ended after the third CT scan because the subjects had reached skeletal maturity (age eight to nine months) revealed by the disappearance of the epiphyseal cartilage of the humerus. The rabbits, previously anesthetized with an intramuscular injection of two ml of two per cent xylazine and 0.3 ml of tiletamine HCl and zolazepam HCl, were euthanized with an intravenous injection of 0.4 ml of embutramide, mebenzoin and tetracaine.

### CT scan measurement

**Figure 1:**
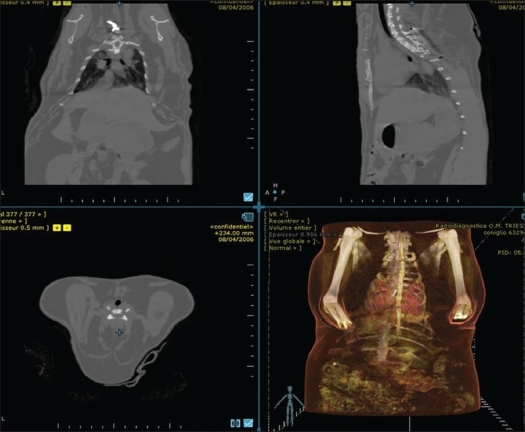
Myrian Pro® software with CT scan images and three dimensional reconstruction of the rabbit body (bottom right image)

Measurement was always carried out by the same operator to avoid interobserver error, by means of the Myrian Pro® software^23^ [Figure 1].

#### a. Thoracic dimensions

Chest depth (Cd^r^), calculated from the ventral face of the vertebral body to the dorsal face of the sternum. Chest width (Cw^r^) connects the inner faces of two symmetrical ribs, lies perpendicularly to Cd^r^ and cuts it in half on the widest point of the thoracic cage. Chest depth and width were then used to obtain the Thoracic index (ThI^r^), expressed by the ratio Cd^r^/Cw^r^ of the thorax

#### b. Thoracic vertebrae bodies dimensions

Dorsal Length (DL^r^), Ventral Length (VL^r^) and Height (HL^r^). Vertebrae dimensions were used to determine the Vertebral Index (VeI^r^), expressed by the ratio DL/VL, and the Vertebral Area (VeA^r^), lying on the mid-sagittal plane of the vertebral body and expressed in square millimetres (mm2) by the formula [(DL^r^+VL^r^)HL^r^]:2.

#### c. Sternum

Sternal Length (StL^r^), calculated in mm from the cranial end of the trachelian cartilage to the caudal end of the xiphoid cartilage.

#### d. Lungs

Static Lung Volume in cm^3^ (V^r^).

### Histological analysis

Coronal and sagittal segments of the thoracic spine were obtained, fixed in formaldehyde, decalcified and prepared for histology. Serial 8-10 *μ*m-thick paraffin sections from fused levels of the arthrodesed rabbits were stained with Hematoxyline and Eosine and Toluidine blue and compared to specimens from nonfused levels and specimens from the controls.

### 2. Clinical study

The specific aim of this study is to describe the thoracic dimensions observed at maturity in patients who had fusion of the thoracic spine prior to age nine and to compare them with those dimensions that would have been expected without the presence of deformity or surgery. Emans *et al*. showed that pelvic inlet width measured by CTs or plain radiographs is an age-independent predictor of the expected thoracic dimensions in unaffected children and adolescents and have published these values.^24^

Twenty one patients who underwent spinal fusion prior to age nine were reviewed. Data on radiographic changes at the preoperative and postoperative examinations were retrospectively evaluated. Antero-posterior and lateral radiographs of the spine including the pelvis were performed in all patients. Preoperative and postoperative radiographic measurement of the spinal curvature in the coronal plane was performed using the Cobb method. Thoracic spine height (Th^c^), lumbar spine height (Lh^c^), trunk height (TRh^c^), pelvic width (Pw^c^), chest width (Cw^c^) and chest depth (Cd^c^) were measured at skeletal maturity and compared to Eman's reference values to assess the predicted thoracic dimensions. Th^c^ and Lh^c^ measurements were obtained by measuring from the upper border of T1 to the lower border of T12 and from the upper border of L1 to the lower border of L5 respectively. TRh^c^ was obtained by the upper border of T1 to the lower border of L5. Pw^c^ was obtained by measuring from the inner cortex of the ilium to the opposite inner cortex of the ilium at the widest portion of the pelvic inlet. Cw^c^ was obtained by measuring the inner portion of the rib to the inner portion of the opposite rib at the widest part of the chest. Cd^c^ was calculated by measuring lateral radiographs of the spine, the distance between the posterior cortex of the sternum and the anterior cortex of the posterior rib at the level of the sixth thoracic vertebra.

## RESULTS

### 1. Experimental arthrodesis

After interpretation of the CT scans at t1, t2 and t3, the 12 rabbits were divided into three groups:

G1, six operated rabbits with correct positioning of the bars and complete fusion;

G2, three operated rabbits with correct positioning of the bars but incomplete fusion;

G3, three shams operated rabbits.

### CT scan measurement

#### a. Thoracic dimensions

The mean values (mm) of chest depth (Cd^r^) and chest width (Cw^r^), – identified on transverse planes of the thorax passing through the corresponding vertebral segments T1-T6 of the spine and the mean values of thoracic index (ThI^r^) show statistical differences between groups for Cd^r^ (P less than 0.05) in CT scans at t2 and t3. In particular, G2 and G3 are similar in their values and always differ from G1. No statistical difference between groups is noted for Cw^r^. In the same way, there are some statistical differences of ThI^r^ (*P* less than 0.05). The ThI^r^ value trend illustrates that in G1 the growth of Cd^r^ is clearly decreasing over time leading to lower values of the index. The mean values of ThI^r^, which are less than one in G1 and G2, prove that Cd^r^ grows less, t from the start and continues to fall further behind the corresponding values of Cw^r^. On the contrary, the values of ThI^r^ in G3 are higher than one showing that Cd^r^ stays above the corresponding values of Cw^r^. In other words, it is possible that Cw^r^ is less influenced by the posterior arthodesis [Table 1].

**Table 1:** Mean and standard deviation (±) ratio, in Gl, G2 and G3 considered separately, of thoracic index (ThI^r^), obtained from CT scans at t1, t2 and t3

	Group	CT at t1 (10 days post surgery) ThI^r^	CT at t2 (55 days post surgery) ThI^r^	CT at t3 (139 days post surgery) ThI^r^
T1-T6	G1	1.00±0.021	0.91±0.053	0.87±0.037
	G2	0.97±0.052	0.97±0.082	0.87±0.253
	G3	1.04±0.035	1.03±0.053	1.03±0.048

#### b. Thoracic vertebral bodies dimensions

The values (mm) of dorsal length (DL^r^) and ventral length (VL^r^) and area (VeA^r^) of individual bodies of thoracic vertebrae identified on sagittal planes of the body of vertebra in the distinct segments T1-T6 and T7-T12 of the spine show statistical differences between groups for DL^r^ and VL^r^ of some vertebrae at t2 and t3 of the segment T1-T6 of the spine (*P* less than 0.05), which was not seen in groups T7-T12. The total dorsal vertebral length (TDL^r^), total ventral vertebral length (TVL^r^) and total vertebral area (TVeA^r^) of the bodies of thoracic vertebrae are expressed by the mean of the sum of individual lengths and surfaces, as reported in Tables 2 and 3. In G1 the TDL^r^, TVL^r^ and TVeA^r^ sums for the segment T1-T6 are statistically significant and less than in G2 and G3, which have similar values at t2 and t3. No statistical significance is noted for the same measures of segments T7-T12.

**Table 2:** Sum (Σ mm) and standard deviation (±), in G1, G2 and G3 considered separately, of dorsal (DL^r^) and ventral (VL^r^) lengths of thoracic vertebral bodies from T1 to T6 and T7 to T12, obtained from CT scans at t1, t2 and t3. Mean (M) of vertebral index (VeI^r^), obtained as mentioned above

		CT at t1 (10 days post surgery)			CT at t2 (55 days post surgery)			CT at t3 (139 days post surgery)		
				
	Group	Σ DL^r^	Σ VL^r^	M VeI^r^	Σ DL^r^	Σ VL^r^	M VeI^r^	Σ DL^r^	Σ VL^r^	M VeI^r^
T1-T6	G1	41.3±1.19	39.7±1.19	1.05±0.93	47.2±0.56^B^	43.8±0.10^B^	1.09±0.07^A^	48.7±0.33^B^	46.5±0.26^B^	1.06±0.06^A^
	G2	42.2±0.35	40.8±0.35	1.04±0.59	48.0±0.12^A^	45.2±0.44^A^	1.07±0.09^A^	51.7±0.38^A^	50.1±0.42^A^	1.04±0.09^AB^
	G3	41.4±0.75	40.1±0.10	1.04±0.58	50.5±0.44^A^	48.7±0.26^A^	1.04±0.04^B^	54.4±0.52^A^	53.8±0.06^A^	1.01±0.04^B^
T7-T12	G1	60.7±1.36	59.5±1.18	1.02±0.03	71.1±1.32	70.0±1.32	1.02±0.02	79.8±1.63	78.4±1.35	1.02±0.02
	G2	61.2±0.40	59.9±0.55	1.02±0.02	70.4±0.50	69.6±0.31	1.01±0.02	79.8±0.87	78.0±0.32	1.02±0.02
	G3	60.2±1.73	58.5±1.39	1.03±0.02	72.8±0.70	71.9±0.65	1.01±0.01	81.3±1.01	80.1±1.04	1.01±0.02

A, B = *P*<0.05

**Table 3:** Sum (Σ mm^2^) and standard deviation (±), in G1, G2 and G3 considered separately, of vertebral area (VeAr) of thoracic vertebral bodies from T1 to T6 and from T7 to T12, obtained from CT scans at t1, t2 and t3, and differences (Δt2-t1 and Δt3-t2)

	Group	CT at t1 (10 days post surgery) Σ VeAr	CT at t2 (55 days post surgery) Σ VeAr	CT at t3 (139 days post surgery) Σ VeAr	Δ t2-t1	Δ t3-t2
T1-T6	G1	124.6±2.8	153.0±3.2	167.5±3.3^B^	28.4^B^	14.5^C^
	G2	128.1±2.7	153.8±3.5	184.0±3.8^B^	25.7^B^	30.2^B^
	G3	129.2±3.5	163.2±3.9	200.6±3.3^A^	34.0^A^	37.4^A^
T7-T12	G1	214.0±.9.3	291.9±13.7	337.0±16.8	77.9	45.1
	G2	219.3±9.4	289.7±13.1	333.7±15.3	70.4	44.0
	G3	216.9±9.5	298.8±12.4	338.7±15.2	81.9	39.9

A, B, C = *P*<0.05

The means of total dorsal vertebral length (TDL^r^), total ventral vertebral length (TVL^r^) and total vertebral area (TVeA^r^ in mm^2^) at different CT scan intervals (t), after the first measurement (Δt2-t1), increase less in G1 and G2 than in G3. After the second measurement (Δt3-t2), TDL^r^, TVL^r^ and TVeA^r^ increase less in G1 than in G2 and G3, which share the same growth rate. It can be inferred, when considering the whole length and area of the study, that the increase in total dorsal length, ventral length and vertebral area of segment T1-T6 is less in G1 and G2 than in G3. No growth differences were found in the spinal segment T7-T12. These differences are supplemented by other information coming from the subsequent calculation of Vel^r^, whose mean values are derived separately for G1, G2 and G3 at t1, t2 and t3 in the distinct segments T1-T6 and T7-T12 of the spine. Vel^r^ that are less close to 1, in G1 and G2 with arthrodesed block, show that DL^r^ and VL^r^ have more pronounced differences between them, which contributes to vertebral irregularity or asymmetry [Tables 2 and 3].

On the contrary, VeI^r^ closer to one, in sham G3 in the T1-T6 segment and in G1-G3 in the T7-T12 segment prove that DL^r^ and VL^r^ in the nonarthrodesed situation do not diverge much from each other. Dorsal kyphosis was reduced in the sement T1-T6 but maintained in the lower T7-T12 segment.

#### c. Sternum

The mean values (mm) of Sternal Length (StL^r^), are 75.4 ± 2.364 (t1), 88.6 ± 4.841 (t2) and 95.1 ± 4.904 (t3) in G1; 83.2 ± 2.001 (t1), 98.0 ± 4.313 (t2) and 103.6 ± 3.400 (t3) in G2; and 81.9 ± 2.471 (t1), 100.3 ± 0.777 (t2) and 107.6 ± 0.473 (t3) in G3. In G1 and G2, sternal length increases less than in G3. G1 is always less than G2 and G3 (*P* less than 0.05). In the same way, the difference between the StL^r^ at different CT scan intervals (Δt) appears less significant in G1 than G2 and G3 at the first interval (Δt2-t1), while at the second interval (Δt3-t2) no difference is shown. In addition, from the difference between sternal lengths at Δt3-t1, it is deduced that in G1 and G2, sternal length increases less than the corresponding sternal length in G3.

#### d. Lungs

The values of static lung volume (V^r^), in cubic centimeters (cm^3^), show remarkable individual differences between the three groups at t1, t2 and t3 and evolve differently. G1 shows an average increase in V^r^ of 1.67 cm^3^ between t2 and t3, and G3 of 8.27, whereas the situation seems to be reversed between t1 and t2, albeit accompanied by a reduction in differences.

### Histological analysis

**Figure 2:**
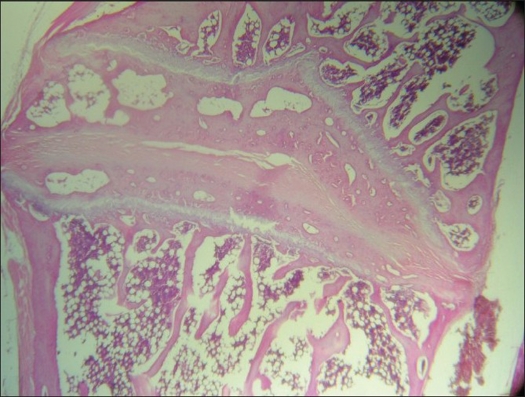
Photomicrograph taken through two adjacent end plates of arthrodesed rabbits depicting disorganization of the annulus fibrosus with some area of ossification of two adjacent end plates

**Figure 3:**
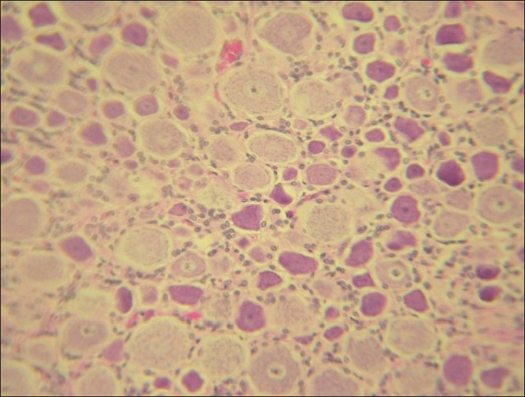
Photomicrograph showing two populations of neurons identified in the root ganglia of arthrodesed rabbits. Type A (normal): homogeneous aspect, circular and regular contour, clear nucleus and round, hyper chromic nucleolus. Nissl substance with typical “dust-like” appearance. Type B (abnormal): irregular contour, acidophilic cytoplasm, Nissl substance granules and picnotic nucleus poorly identifiable. Satellitosis of the perineuronal cells

Arthrodesed rabbits showed disorganization of the annulus fibrosus with some areas of ossification connecting adjacent end plates [Figure 2]. Intervertebral joint and costovertebral joints showed cartilage degeneration and loss of congruence but no sign of ossification or fusion. Increased vascularization was found in surrounding tissues. Two populations of neurons were identified in the root ganglia of arthrodesed rabbits: one normal and one with abnormal changes. These changes included neurons with smaller and irregular perykarion, abnormal shape and atypical Nissl substance distribution. Additionally, the architecture of the satellite cells sheath appeared disorganized [Figure 3]. The radicular motor neurons showed homogeneous Nissl substance distribution and the spinal cord appeared overall normal.

#### Clinical study

Eleven female and 10 male patients were included. The mean age of the patients at time of surgery was 5.9 ± 2.46 years. All patients had reached skeletal maturity at the last follow-up visit and the average follow-up was 11.6 years. Eleven patients (52.4%) had congenital scoliosis, five (23.8%) had neuromuscular scoliosis, and three (14.3%) had scoliosis secondary to genetic syndrome and two to neurofibromatosis type-1 (9.5%). Nine patients (42.9%) underwent posterior spinal fusion with or without instrumentation and 11 (57.1%) had a combination of anterior and posterior surgery. The spinal instrumentation varied accordingly to underlying pathology. Standard segmental instrumentation using dual rods and hooks was used for seventeen patients and segmental instrumentation into the pelvis (unit rod or Luque rod) for four patients. The average number of spinal segments fused was 10 ± 3.9. At skeletal maturity the mean Th^c^ was 20 ± 6.1 cm for boys and 19 ± 5.2 cm for girls; the mean Lh^c^ was 13.8 ± 2.6 cm for boys and 13.2 ± 3.5 cm for girls. The mean Pw^c^ was 12.7 ± 1.6 cm for boys and 12.2 ± 2.0 cm for girls; the mean Cw^c^ was 24 ± 2.8 cm for boys and 22.1 ± 3.3 cm for girls and the mean Cdc was 8.7 ± 1.1 cm for boys and 9.3 ± 1.6 cm for girls [Table 4]. According to Dimeglio *et al*, the thorax has an average depth of 21 cm in boys and 17.7 cm in girls and an average width of 28 cm and 24.7 cm respectively at the end of growth. 25, 26

**Table 4:** Gender curve magnitude in degrees, age at fusion in years, and observed PW^c^, CW^c^, CD^c^, Th^c^, Lh^c^, and Th^c^/Lh^c^ in male and female patients

Pt.	Sex	Curve	Age at fusion	PW^c^	CW^c^	CD^c^	Th^c^	Lh^c^	Th^c^/Lh^c^
1	F	T4-T10; 75°	8.2	14.3	21.8	9.2	20.6	15.7	1.3
2	F	T2-T7; 60°	7.9	14.1	26.8	6.3	25	14.8	1.7
3	F	T6-T12;	6.9	14	21.6	9.6	15.7	11	1.4
4	F	T1-T7; 58°	1.2	12.6	27.6	10.7	23.8	11.3	2.3
5	F	T5-T12; 77°	8.2	13.7	16	8.8	17.3	12.1	1.4
6	F	T4-T9; 60°	7.3	13.2	21.4	11.3	20.9	17.7	1.2
7	F	T7-T9; 32°	1.3	12.7	24.7	10.5	25.8	18.8	1.4
8	F	T7-L3; 71°	7.9	10.3	20.1	8.8	17.1	13	1.3
9	F	T8-L3; 58°	5	11.1	19.9	8.1	18.9	8.4	2.2
10	F	T3-L3; 98°	2.5	10.1	20.1	7.6	7.7	8.1	0.9
11	F	T5-L3; 62°	6.1	8	23	9.1	22.6	14	1.6
12	M	T6-L5; 156°	7.9	11.4	21.6	8.5	18.3	11.2	1.6
13	M	T9-L3; 84°	5.3	12.6	25.6	8.2	15.1	9.5	1.6
14	M	T5-L1; 58°	6.9	12.1	22.1	7.1	14.6	13.8	1.0
15	M	T8-L2; 73°	8.3	12.2	25.4	7.1	21.2	14.5	1.5
16	M	T1-T9; 58°	5.4	12.3	26.4	10.5	22.3	17.8	1.2
17	M	T6-L1; 79°	3.3	14.6	21.4	8.5	17	15.5	1.1
18	M	T5-L1; 104°	7.9	16.3	24.5	9.1	24.8	14.4	1.7
19	M	T5-L1; 60°	6.5	11.1	19.6	8.2	17	11.6	1.5
20	M	T6-L1; 80°	8.2	12.4	29.2	10.1	34.5	12.5	2.8
21	M	T2-T12; 63°	1.9	11.6	24.4	8.7	15.4	16.9	0.9

(M = Male; F = Female),

In our group of patients, thoracic depth and width were both significantly reduced. However, depth was more severely affected than width and the chest became flat and elliptical. From previous data the ratio between chest width and depth is 0.75 in both boys and girls. In our group, the ratio dropped to 0.4 suggesting a more severely reduced Cd^c^ growth.

Pulmonary functions tests were done on 10 of the 21 patients (47.6%). The average forced vital capacity was 1.3 liters and average forced expiratory volume in one second was 1.07 liters. Most of the patients developed restrictive lung disease.

## DISCUSSION

Both the experimental and clinical study reported in this work were intended to evaluate how spinal arthrodesis done at an early stage of development affected chest, vertebrae, sternum and neural elements. In humans, definitive spinal fusion can realign the spine but it may result in a short trunk and disproportionate body habitus. On average, the newborn thoracic perimeter is 32.3 cm in boys and 31.5 cm in girls and it will attain a mean value of 89.2 cm and 85.4 cm respectively. The overall chest shape evolves from ovoid at birth to elliptical at skeletal maturity. This suggests that thoracic width increases more than depth during normal growth. According to Dimeglio *et al*, at the end of growth the thorax has an average chest depth of 21 cm in boys and 17.7 cm in girls and an average chest width of 28 cm and 24.7 cm respectively.^22^,^23^ In our studies, both thoracic depth and width were significantly reduced. However, depth was more severely affected than width and the chest became flat and elliptical in shape. From previous data the ratio between chest depth and width is 0.75 in both boys and girls. In our group of patients the ratio dropped to 0.4 suggesting a more severely reduced Cd^c^ growth.

Karol *et al*, have shown that spinal arthrodesis in young children reduces thoracic depth and shortens the T1-T12 segment. Fusion is a cause of respiratory insufficiency and adds to the spinal deformity the loss of pulmonary function.^27^

Recently, in a growing rabbit model, an investigation into the association of growth of the spine and thorax under conditions that create growth disturbances of the spine or thorax showed that a unilateral deformity of the spine or thorax induces both scoliosis and thoracic cage deformity with asymmetric lung volumes. In our study, the analysis of V^r^ for partial experimental times shows a slowing down in the growth of V^r^ (+ 1.67 cm^3^) in G1 between CT scans taken at t2 and t3, as opposed to a more pronounced increase (+ 8.27 cm^3^) in G3. The reduced increase in V^r^ could still be observed even when not the entire thoracic spine was blocked by dorsal arthrodesis: this observation could suggest that thorax development is a process involving various parts in a uniform and synchronous manner, including the 12 sternal and asternal ribs which together maintain the same growth rate, and influence one another. Blocking approximately 50% of the thoracic spine and especially blocking those vertebrae which, through sternal ribs, are articulated with the sternum, would also affect the lower or following rib levels and would therefore lead to a reduced total V^r^, due in turn to a decreased capacity of the thoracic cage. At the same time, however, reduced thorax growth does not seem to fully involve vertebral growth because in the presence of dorsal arthrodesis, thoracic vertebrae in the T1-T6 segment show growth irregularities, whereas those that follow in the T7-T12 segment, which are articulated with asternal ribs and are not blocked by dorsal arthrodesis, maintain a normal growth pattern. Thoracic vertebrae T1-T6 of operated rabbits show an overall growth of DL^r^ and VL^r^, and of VeA^r^, which is reduced by approximately 12% and 16% compared with thoracic vertebrae T1-T6 of sham subjects, whereas the T7-T12 segment grows with differences lower than two per cent and 0.5% between the three groups.

Assuming that the spine can be likened to two connected bone columns, one anterior or ventral and one posterior or dorsal, Crankshaft phenomenon is the progression of deformity when the growth of the anterior column persists, whereas the posterior column is blocked by arthrodesis.^2^,^28^,^29^ To avoid the occurrence of Crankshaft phenomenon, posterior vertebral arthrodesis should not be carried out in skeletally immature patients. Quadrupeds, on the contrary, do not often develop scoliosis, and therefore the equivalent of Crankshaft phenomenon is the reduction of kyphosis after dorsal vertebral arthrodesis. The irregular growth of vertebral bodies is the basis of a distorted development, both in human bipeds and in quadrupeds. Coleman^4^ and Veliskakis^3^ showed it in dogs, Ottander^30^ in pigs. Kioschos,^31^ on skeletally immature dogs, confirmed that the presence of dorsal vertebral arthrodesis leads to a significant reduction of dorsal kyphosis, even without implanted material, as a consequence of the ventral growth of vertebral bodies – which therefore, the author says, become asymmetrical. Similar findings were reported by Moon *et al*, who demonstrated that instrumented static posterior compression of dog's growing spine by hooked rods, suppresses the posterior spinal growth more than the anterior.^14^

The present study compared the T1-T6 segment of the cranial portion of the spine, where the effects of vertebral fusion became manifest, with the T7-T12 segment of the caudal portion. With arthrodesis of the cranial portion of the spine, irregularity in the growth of vertebral bodies occurred as lower values of DL^r^, VL^r^, VeA^r^, and VeI^r^ demonstrates. Thoracic vertebrae T1-T6 of operated subjects show a diminished growth of DL^r^ and VL^r^. VeA^r^ is reduced by approximately 14% compared with thoracic vertebrae T1-T6 of sham subjects, whereas the T7-T12 segment grows with differences lower than 2% between the three groups. Similarly, our clinical study confirmed that both T1-T12 and L1-L5 segments grew less than expected when were included in the fusion instrumentation.

Rabbits' sternum development was also affected. The sternum grew less since it is effectively in articular continuity with the T1-T6/7 segment through the sternal ribs. The ventral portion of the spine, which corresponds to the vertebral bodies, continues to grow, while the dorsal portion, which corresponds to the laminae, ceases to grow: the result, in our “rabbit model”, is a reduction of its dorsal kyphosis. Quadrupeds do not often develop scoliosis, and therefore the equivalent of Crankshaft phenomenon is the reduction of kyphosis after dorsal vertebral arthrodesis.^3^,^4^,^12^–^14^,^24^

Histological findings suggest a possible interaction between surgery, spine and neural elements.^32^ Arthrodesed rabbits showed disorganization of the annulus fibrosus with ossification of two adjacent end plates which lead to spontaneous anterior fusion. Those findings, however, do not match the findings of Moon *et al*, who evaluated the effects of posterior spinal fixation with acrylic cement on the vertebral growth plate and intervertebral disc in dogs. The authors reported thinning and disorganization of the growth plate but not growth arrest.^12^,^13^,^16^

## CONCLUSION

Development of the thoracic cage and lungs is a complex process which requires perfect synergy among the various components of the thoracic cage. Alterations in some of these elements affect and change the development and growth of the others. Both the experimental and the clinical studies intended, first, to evaluate the consequences of a disturbed growth of vertebral bodies on the development of the other elements, i.e. ribs, sternum, and lungs, which are part of the thoracic cage. A cause-effect relationship can be proposed between the arthrodesis of the cranial segment of the thoracic spine and the reduced growth of the thorax when the dorsal arthrodesis is performed in young and very young patients. In this case, the effects on the growth of the thorax, with a lower growth of chest depth changing from a fairly circular shape to one that tends to be elliptical, are particularly evident.

To preserve thoracic motility and permit a normal development of the respiratory tree, the treatment, without focusing only on the spine, should consider the thorax as a whole. Several techniques are available and possible: surgical techniques such as growing rods and/or thoracic expansion and non-surgical techniques such as traction, serial casts and braces which postpone surgery.
